# Naloxone precipitated withdrawal increases dopamine release in the dorsal striatum of opioid dependent men

**DOI:** 10.1038/s41398-021-01548-8

**Published:** 2021-09-01

**Authors:** Ehsan Shokri-Kojori, Gene-Jack Wang, Nora D. Volkow

**Affiliations:** Laboratory of Neuroimaging, National Institute on Alcohol Abuse and Alcoholism, National Institutes of Health, Bethesda, MD USA

**Keywords:** Addiction, Molecular neuroscience

## Abstract

Dopamine (DA) neurotransmission is critical in the neurobiology of reward and aversion, but its contribution to the aversive state of opioid withdrawal remains unknown in humans. To address this, we used updated voxelwise methods and retrospectively analyzed a [^11^C]raclopride-PET dataset to measure D_2/3_ receptor availability and relative cerebral blood flow (R1) in male opioid use disorder (OUD) participants (*n* = 10) during placebo and acute opioid withdrawal conditions. We found that acute withdrawal precipitated by the opioid antagonist naloxone significantly increased dorsal striatal DA release in OUD participants (*p*_FWE_ < 0.05). Net changes in striatal DA were significantly correlated with a subjective index of withdrawal aversion such that greater DA increases were associated with more aversive responses (*r*(8) = 0.82, *p* < 0.005). Withdrawal also affected brain function, as indexed by increases in relative cerebral blood flow in the insula and putamen (*p*_FWE_ < 0.05). Our findings are different from preclinical studies that have primarily reported decreases in ventral striatal DA during naloxone precipitated withdrawal, whereas this effect was not significant in OUD participants (*p* = 0.79). In sum, we provide evidence for the contribution of increases in dorsal striatal DA to the aversive state of naloxone precipitated withdrawal in humans.

## Introduction

The opioid epidemic is a leading health crisis in the US. Acute opioid withdrawal is an important contributor to drug-seeking behavior and relapse, and its underlying neurobiology is critical for the treatment development of opioid use disorder (OUD). Changes in opioid neurotransmission contribute to physical dependence and withdrawal symptomatology in OUD [1, 2]. Changes in the dopamine (DA) system, the main contributor to reward and aversion [3, 4], have been also implicated in OUD. Similar to other substance use disorders [5], OUD has been associated with decreased striatal D_2/3_ receptor (D_2/3_R) availability [6, 7]. Opioids, directly and indirectly, affect midbrain DA neurons in the ventral tegmental area (VTA) and substantia nigra pars compacta (SNc). Stimulation of mu-opioid receptor (MOR) suppresses the inhibitory effect of GABA interneurons on DA neurons in the VTA. This indirect inhibition results in increased DA release in the ventral striatum that has been associated with the rewarding effects of opioid drugs [8]. Kappa opioid receptors (KORs) also regulate DA release through their direct effect on DA neurons and are implicated in aversion including opioid withdrawal [9, 10]. Different effects of opioid antagonists and agonists on the opioid receptors in the SNc and VTA could differentially affect DA release in the dorsal and ventral striatum [11].

Symptoms of acute opioid withdrawal primarily reflect disruptions in opioid signaling in multiple neuronal systems following repeated opioid exposure. Preclinical data indicate that acute opioid exposure stimulates MOR in the locus coeruleus (LC), suppresses norepinephrine release, and reduces adrenergic function [12]. Chronic opioid exposure increases basal LC activity, presumably, to counterbalance the repeated suppression of norepinephrine release [13]. Abrupt discontinuation of opioids or administration of opioid antagonists (such as naloxone (NAL)) markedly increases norepinephrine release and triggers systemic withdrawal symptoms [14]. Acute opioid withdrawal is stressful and stimulates the hypothalamus–pituitary–adrenal (HPA) axis, leading to increases in pituitary pro-opiomelanocortin mRNA, adrenocorticotropic hormone (ACTH), and adrenal cortisol release [15].

NAL, a non-selective opioid antagonist [16, 17], is used to reverse opioid overdose effects [18] and precipitates withdrawal in opioid-dependent individuals. Here we aimed to study the contribution of DA to NAL precipitated withdrawal (NPW) in OUD participants. For this purpose, we employed updated voxelwise PET analysis techniques (to improve image co-registration, normalization, and reference region identification) and revisited a [^11^C]raclopride-PET dataset where we had assessed changes in DA release between placebo and NPW conditions in OUD men [6]. As a secondary goal, we explored the functional effects of NPW (indexed by relative cerebral blood flow, rCBF) on the pituitary (related to stress and HPA stimulation) [15, 19] and habenula (implicated in NAL aversion) [20]. Preclinical studies that assessed the effect of NPW on DA, have mostly focused on the ventral striatum and have reported decreases [10, 21–25] or no change in DA release [26]. In contrast, increases in DA have been reported in the dorsal striatum during NPW [27]. Thus, we hypothesized that NPW would increase DA in the dorsal striatum and decrease DA in the ventral striatum and that changes in striatal DA would be associated with a subjective index of withdrawal aversion.

## Methods

### Participants

This study entailed a retrospective analysis of a [^11^C]raclopride-PET brain imaging dataset [6] with a total of 20 PET scans that were collected on 10 OUD participants (10 males, age = 40.8 ± 4.3 years) in two sessions following administration of saline (SAL) and NAL (see PET imaging and modeling). The original report [6] included 11 OUD participants with PET data collected during SAL, out of which 9 OUD participants were reported to undergo [^11^C]raclopride-PET imaging during NAL condition. However, after reexamination of data records, we were able to identify an additional OUD participant with [^11^C]raclopride-PET data for both sessions (a total of 10 OUD participants). The remaining OUD participant with [^11^C]raclopride-PET data collected only under SAL condition was not included in this report. OUD participants were studied at Brookhaven National Laboratory and provided written informed consent that was approved by the Human Subjects Research Committee of the Brookhaven National Laboratory. OUD participants had at least one year history of continued opioid use (heroin or methadone treated) and met DSM-IV criteria for opioid dependence. OUD participants were also current smokers (*n* = 8), current marijuana users (*n* = 7), current (*n* = 4) or past (*n* = 3) cocaine users, current (*n* = 2) or past (*n* = 1) amphetamine users, and past LSD users (*n* = 2). The sample size was justified based on a recent study showing that the effect of morphine on striatal DA could be detected in 10 healthy controls [28]. Imaging data on two healthy controls were also available [6] but not included in this analysis due to the small sample size.

### PET imaging and modeling

The OUD participants (*n* = 10) underwent two consecutive dynamic [^11^C]raclopride-PET scans (CTI-Siemens ECAT 931, ~6 mm FWHM) on the same day, except for two participants who were studied on different days. The first scan with [^11^C]raclopride started 5–7 min following administration of intravenous (IV) SAL. After 2 h, the second scan with [^11^C]raclopride was started following administration of IV NAL that was given in 0.01 mg/kg increments (every 4 min) until withdrawal symptoms appeared (e.g., yawning, abdominal cramps, rhinorrhea, and tearing) [6]. OUD participants received about 0.02 mg/kg of NAL. Two participants received 0.01 mg/kg NAL and were scanned on a different day from the SAL scan. Frame-wise motion correction was applied to all scans. Both dynamic [^11^C]raclopride-PET scans for each participant were co-registered and were normalized to MNI space (2-mm isotropic) using FSL (the FMRIB Software Library) [29] and AFNI [30] routines. To estimate D_2/3_R availability in OUD, dynamic [^11^C]raclopride-PET data were modeled in PMOD 3.9 (PMOD Technologies Ltd, Zurich, Switzerland). The simplified reference tissue model 2 (SRTM2) [31] with a cerebellar reference region was used to estimate voxelwise binding potential (BPnd) and *R*1 (*R*1 = *K*1/*K*′1). *R*1 is a proxy of rCBF with good agreement with rCBF measures obtained by [^15^O]H_2_O-PET [32–34] and with high reliability for [^11^C]raclopride-PET [35].

### Voxelwise comparisons

Voxelwise paired *t*-tests were performed to assess NAL effects on [^11^C]raclopride BPnd and R1 in SPM12 (Wellcome Trust Centre for Neuroimaging, London) [36]. Images were smoothed with a 5-mm full-width at half-maximum Gaussian kernel prior to the paired *t*-test analyses. A threshold of *p* ≤ 0.01 in SPM12 was used and cluster sizes were corrected for multiple comparisons in the whole brain using the random field theory to control family-wise error (*p*_FWE_ < 0.05) [37].

### Net DA release and net rCBF change

DA release was calculated by subtracting the D_2/3_R BPnd in the drug condition (e.g., NAL) from that in the SAL condition and dividing the result by D_2/3_R BPnd in the SAL condition (to normalize for regional differences in D_2/3_R). Net DA release was calculated by averaging DA release across the striatum. To estimate drug-induced changes in rCBF, we subtracted R1 in the SAL condition from that in the drug condition and divided the result by R1 in the SAL condition (to normalize for regional differences in R1). Net rCBF change was calculated by averaging rCBF change across the striatum. When R1 measures are directly being compared, we use R1 in the text, otherwise, we use rCBF change or net rCBF change as defined here.

### Regions of interest (ROIs)

We assessed NPW-induced changes in [^11^C]raclopride BPnd using a priori ROIs of the dorsal (caudate and putamen combined) and ventral striatum [38]. We also performed exploratory analyses to assess the effect of NPW on R1 in the pituitary and habenula ROIs. The pituitary has high MOR levels [39, 40], is a major source of endorphins (endogenous MOR agonist), and modulates the HPA response during NPW. Increases in pituitary R1 (measured with PET) have been observed in conditions that trigger the release of pituitary hormones [41]. For this purpose, we used T1w (available in FSL) and magnetic resonance angiography (MRA) maps [42] in the MNI space with 2-mm isotropic resolution to extract anatomical ROIs for the pituitary gland and the nearby internal carotid arteries. The pituitary ROI was centered at (MNI: *x* = 0 mm, *y* = 2 mm, *z* = −32 mm) [43] and expanded to cover nearby areas with high T1w image intensity (63 voxels, 0.50 mL). We used high-intensity areas in the MRA map, adjacent to the pituitary ROI, to create a bilateral internal carotid ROI (666 voxels, 5.33 mL). The pituitary and carotid ROIs had no spatial overlap. The habenula has notably high levels of MOR (medial habenula) [44] and also expresses KOR (lateral habenula) [45], is implicated in aversive states [46] including NPW [20], and modulates DA release [47, 48]. A bilateral habenula ROI was defined based on MNI coordinates defined in the literature [49]. An adjacent control ROI was positioned anterior to the habenula ROI. We tested the effect of NPW on R1 in both bilateral ROIs and corrected for multiple comparisons for these two ROIs (*n* = 2, *p* < 0.025). Considering that the volume of the habenula was only a fraction of the spatial resolution of the PET camera, we were unable to distinguish between LHb and medial habenula (the segment implicated in NAL aversion) [20], and our habenula findings should be considered preliminary and interpreted with caution.

### Physiological monitoring in OUD

Plasma levels of cortisol and prolactin, cardiovascular measures of systolic and diastolic blood pressure, and pulse rate were recorded during SAL and NAL imaging sessions according to procedures described previously [6]. To correct for multiple comparisons across physiological and subjective measures, we applied a familywise false discovery rate of 0.05 [50].

Plasma cortisol and prolactin levels were measured before, and 10, 30, and 60 min after SAL and NAL injection in 9 OUD participants but complete data was only available at 30 and 60 min post-injection. We averaged plasma levels at these later time points and compared the average cortisol and prolactin levels between SAL and NAL conditions.

Electrocardiographic and blood pressure recordings were obtained continuously throughout the NAL and SAL sessions (every 1 min for 20 min and then at 25, 30, 45, and 60 min). We averaged cardiovascular data points across each session and compared the changes in cardiovascular measures between NAL and SAL sessions.

### Behavioral monitoring in OUD

Subjective withdrawal experience was measured by self-reports of alertness, anxiety, annoyance, loss of control, depression, distressful thoughts, happiness, mood, restlessness, concentration, desire to use an opiate, optimism, activeness, indifference, irritability, and pain that were scored from 0 for lowest to 10 for highest. These self-reports were obtained 10 min pre (baseline) and 10, 30, and 60 min post-injection in each session (Supplementary Table 1). Additional measures of withdrawal symptoms (e.g., muscle twitches or shivering) were not included here due to their limited variability across participants. The subjective NAL effect was studied by comparing each of the three post-injection data points with the baseline. To estimate an index of NAL aversion, we calculated the first principle component of the (*z*-scored) changes across any of the subjective measures/time points that showed a significant NAL effect (*p* < 0.05, uncorrected). This component accounted for 37% of the variance of changes in these subjective measures and was used as an index of NAL aversion (see Supplementary Table 1), where higher component scores were associated with more aversion.

## Results

### Effects of NPW on striatal DA release

**Fig. 1:**
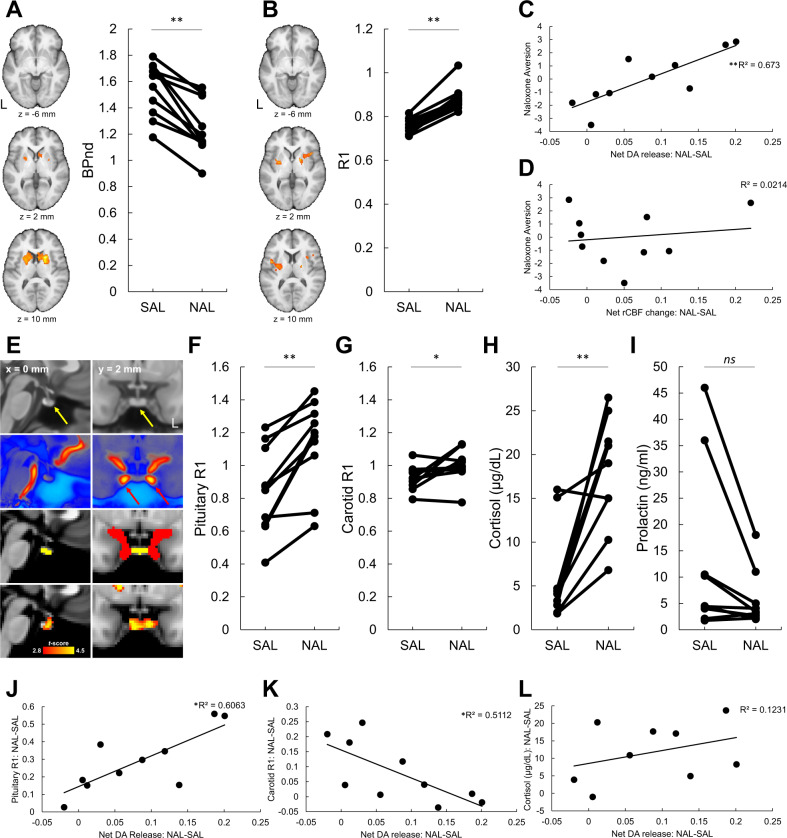
Naloxone precipitated withdrawal affects dopamine release and relative cerebral blood flow in OUD men. **A** Voxelwise *t*-test on [^11^C]racloprideBPnd revealed consistent decreases in NAL compared to SAL (*p*_FWE_ < 0.05). **B** Increases in R1 (a surrogate for rCBF) in the NAL compared to SAL (*p*_FWE_ < 0.05). **C**, **D** Index of naloxone aversion (higher component scores indicate more aversion) was significantly associated with net increases in striatal DA-release but not with net increases in striatal rCBF (see the “Methods” section). **E** Left and right columns show sagittal and axial views of the human brain. First row: T1w map of human brain (0.5-mm isotropic). The yellow arrows point at the pituitary. Second row: magnetic resonance angiography map of human brain (0.5-mm isotropic) [42]. The red arrows point at the internal carotid arteries. Third row: pituitary (yellow) and internal carotid arteries (red) ROIs. Fourth row: map of *t*-scores for the effect of NPW on R1 (cluster size > 200 voxels). **F** Increases in pituitary R1 across participants with NAL compared to SAL. **G** Increases in carotid R1 with NAL compared to SAL. **H** Increases in plasma cortisol with NAL compared to SAL. **I** Changes in plasma prolactin with NAL compared to SAL. It was notable that the two OUD participants with hyperprolactinemia showed a large reduction in prolactin with NAL compared to SAL. **J** Association between net striatal DA-release and increase in pituitary R1. **K** Association between net striatal DA-release and increase in carotid artery R1. **L** Association between net striatal DA-release and increase in plasma cortisol level. *p < 0.05, **p < 0.005.

A voxelwise paired *t*-test showed that NPW significantly increased DA in a cluster including the caudate and putamen of OUD participants, as measured by decreases in [^11^C]raclopride BPnd (*p*_FWE_ < 0.05, Fig. 1A, Supplementary Table 2). For this cluster, the mean ± standard deviation of [^11^C]raclopride BPnd for SAL condition was 1.54 ± 0.21 and for NAL condition was 1.25 ± 0.21. The net increase in striatal DA release (averaged throughout the striatum, see the “Methods” section) was also significant during NPW (*p* = 0.009, Cohen’s *d* = 1.05). Follow-up ROI analyses showed significant NPW-induced decreases in BPnd in the dorsal striatal ROI (SAL BPnd: 1.93 ± 0.27, NAL BPnd: 1.73 ± 0.23, *p* = 0.005) whereas no significant change in BPnd was found in the ventral striatal ROI (SAL BPnd: 1.47 ± 0.26, NAL BPnd: 1.46 ± 0.28, *p* = 0.79). NPW-induced decreases in BPnd in the dorsal striatal ROI were significantly different from changes in BPnd in the ventral striatal ROI (*p* = 0.002).

### Effects of NPW on rCBF

A voxelwise paired *t*-test showed that NPW increased R1 (indexing rCBF) in insular and putamen regions (*p*_FWE_ < 0.05, Fig. 1B, Supplementary Table 3). There was a trend increase in net striatal rCBF (the whole striatum, see the “Methods” section) during NPW (*p* = 0.06) though changes in the dorsal (*p* = 0.11) and ventral (*p* = 0.67) striatal ROIs were not significant. Changes in net striatal DA were not significantly associated with changes in net striatal rCBF (see the “Methods” section) (*r*(8) = 0.005, *p* = 0.99).

### Effects of NPW on subjective measures of withdrawal

NPW was aversive and resulted in significant increases in self-reports of *anxiety*, *annoyance*, and *restlessness* and decreases in *happiness* and *mood* (*p*_FDR_ < 0.05, Supplementary Table 1). A withdrawal aversion index was calculated using the first principal component of the change in subjective measures that showed a significant NAL effect (see the “Methods” section) where higher component scores were associated with more aversion. The withdrawal aversion index was significantly and positively associated with changes in net striatal DA (*r*(8) = 0.82, *p* = 0.0036, Fig. 1C) but not with net changes in striatal rCBF (*r*(8) = 0.14, *p* = 0.69, Fig. 1D).

### Effects of NPW on peripheral measures

NPW elevated plasma cortisol levels and elevated cardiovascular activity (Fig. 1H, *p* = 0.003, *p*_FDR_ < 0.05, Supplementary Fig. 1). Changes in plasma prolactin were not significant (*p* = 0.07, Fig. 1I). The positive correlation between changes in plasma cortisol and changes in net striatal DA was not significant (*r*(7) = 0.49, *p* = 0.15, Fig. 1L).

### ROIs analyses of R1

NPW significantly increased R1 in the pituitary (*p* = 0.0006) and carotid (*p* = 0.03) ROIs (Fig. 1E–G, see the “Methods” section). Net increases in striatal DA was significantly and positively associated with increases in pituitary R1 (*r*(8) = 0.78, *p* = 0.008, Fig. 1J) but significantly and negatively with an increase in carotid R1 (*r*(8) = −0.71, *p* = 0.02, Fig. 1K). NPW also increased R1 in the habenula ROI (*p* < 0.025, see Supplementary Fig. 2 and the section “Methods”).

## Discussion

Here we document that NPW in OUD participants was associated with increased DA release in the dorsal but not ventral striatum. Increases in striatal DA release were positively associated with withdrawal aversion and with increases in pituitary R1. The findings implicate DA in the aversive experience of acute opioid withdrawal and in the concomitant activation of the HPA axis. Preclinical studies that have measured DA in the ventral striatum have shown that opioid withdrawal is associated with decreases in DA [10, 21–25, 51–54], whereas some studies have reported increases in DA particularly in the dorsal striatum [27, 55]. We found that NPW increased DA in the dorsal striatum and the effect was significantly different from changes in the ventral striatal DA in OUD participants. However, different from the preclinical literature, we did not observe DA decreases in the ventral striatum, which could reflect differences in experimental procedures or interspecies differences.

### DA increases in dorsal but not ventral striatum during NPW

The mechanisms responsible for the DA increases in the dorsal but not ventral striatum during NPW in OUD are unclear and could reflect differences in the effects of NAL on the SNc and VTA. Since NAL is a non-specific antagonist, the regional effect of NAL could be determined by the relative involvement of different opioid receptors in regulating DA neurons and DA release. For example, blockade of KOR (expressed on DA neurons and their terminals) could increase DA release [56, 57]. This effect could be counteracted by MOR blockade which enhances the inhibition of DA neurons (decreases DA release), particularly in the VTA with higher MOR-expressing GABAergic interneurons than SNc [58]. Thus, the less inhibitory effects of MOR blockade in the SNc relative to VTA could account for relative increases in DA release in the dorsal striatum during NPW. In addition, MOR-expressing GABAergic afferents (e.g., from RMTg) interact with midbrain GABAergic interneurons and could lead to MOR antagonist-induced DA release [59]. These mechanisms of DA regulation may have been differently affected in the SNc and VTA by chronic exposure to opioids [60], which could lead to differences in DA release in the dorsal and ventral striatum during NPW. It is noteworthy that dorsal striatal DA increases during NPW were different in the location from the effect of morphine in healthy controls where DA release increased primarily in the ventral striatum [28].

### Aversion-related increases in striatal DA

NPW-induced aversion was associated with net increases in striatal DA, such that OUD participants with more aversive withdrawal experiences had more DA increases. In general, DA increases, particularly in the nucleus accumbens of the ventral striatum, have been observed in response to reward and reward expectation (including drugs) [61, 62] but also aversion and stress [63, 64]. In this respect, distinct subgroups of DA neurons with different afferent projections have been implicated in processing of reward and aversion [48]. Specifically, lateral VTA neurons encode reward and receive excitatory inputs from laterodorsal tegmentum and inhibitory inputs from the lateral habenula (LHb) via RMTg [48, 65]. Medial VTA neurons encode aversion and receive glutamatergic inputs from the LHb [48]. Similar to VTA, different subgroups of SNc DA neurons are implicated in aversion and reward [66]. Thus, aversion-related increases in dorsal striatal DA during NPW may be consistent with excitation of dorsolateral SNc DA neurons that project to the dorsal striatum and respond to aversive stimuli [66, 67]. The lateral habenula is a key projecting region to DA neurons and has a major role in the neurocircuitry of aversion with its indirect inhibitory control [47, 68, 69] and direct excitatory control over the VTA and SNc [70, 71]. LHb is also modulated by opioids through KOR [45] and MOR [72]. Interestingly, though preliminary in nature, we documented a significant increase in rCBF in the habenula during NPW (Supplementary Fig. 2) that supports its involvement in acute opioid withdrawal in humans.

### NPW effects on striatal function

We found a significant regional increase in R1 in the insula and putamen (Fig. 1B) and a trend increase in net striatal rCBF during NPW. However, the correlation between changes in net striatal DA and net striatal rCBF was not significant. Recent reports also revealed a mismatch between amplitude of striatal DA release and BOLD response (related to blood flow and a marker of brain function) [73]. Together with DA, glutamate and aspartate signaling [74] and GABAergic and cholinergic interneurons [75, 76], are expected to modulate striatal function during NPW. Thus, it is likely that striatal rCBF changes during NPW reflect contributions from multiple neurotransmitters [77].

### HPA hyperactivity during NPW

We observed significant increases in plasma cortisol and in pituitary R1 (see the “Methods” section) that were consistent with HPA hyperactivity during NPW [78]. Opioid and DA receptors modulate HPA function [79], for example, MOR is highly expressed in the pituitary [40]. The positive association between increases in pituitary R1 and striatal DA release could reflect concomitant effects of NAL on the pituitary and DA systems [80, 81]. Previous studies have reported positive associations between striatal DA release and cortisol levels during an IV amphetamine challenge (which was generally rewarding) [82] and during an aversive psychological stressor condition [64]. This association was not significant in our study (*r*(7) = 0.49, *p* = 0.15) which could reflect the chronic state of stress in OUD [83] or our limited sample size.

### Limitations and considerations

In a prior analysis of this dataset [6], the failure to detect striatal DA increase during NPW is likely due to limitations in imaging analysis tools. The old analysis did not rely on alignment of sessions to a common anatomical reference which could have been introduced within and between-subject variability in the hand-drawn ROIs. In addition, the striatal ROIs were large (spanning multiple planes) [6] which may have obscured focal effects within striatal subregions. The present study overcame these limitations by aligning participants’ scans and then transferring the images to a common MNI space to identify striatal and cerebellar ROIs and to assess NPW effects on striatal DA at the voxel level. Different from the old analysis, we used a simplified reference tissue model (see the section “Methods”), which does not rely on arterial input function, an approach that is potentially beneficial for eliminating another source of variability (i.e., measurement errors in arterial input function) in the kinetic modeling of PET data [84]. The main limitation of this study is the small sample size (*n* = 10). The difficulty of conducting imaging studies in OUD individuals during acute opioid withdrawal did not allow us to expand the sample size. Yet, we showed a significant and relatively large effect of NPW on striatal DA (Cohen’s *d* = 1.05) that could be clinically meaningful and merits further investigation. However, the behavioral associations with NPW-induced DA release should be interpreted with caution in our small sample. We also cannot ascertain a causal relationship between NPW-induced DA release and withdrawal aversion. Finally, because of the limited PET resolution, ROI analyses in small regions (e.g., habenula) should be considered preliminary.

## Conclusion

Preclinical studies have shown increases or decreases in DA release or neuronal activity primarily in the ventral but also dorsal striatum in response to aversion and reward [59, 61, 63, 85, 86]. In humans, brain imaging studies have reported different BOLD activation in the dorsal and ventral striatum with aversive and rewarding stimuli [63, 87–89]. Other than a prior study showing that psychological stress increased striatal DA release in the human ventral striatum [64], our findings are the first to document in humans that a drug-induced aversive and stressful state was associated with DA increases in the dorsal striatum. We also showed that the striatal DA increases were associated with the severity of opioid withdrawal experience. These observations support the contribution of DA to opioid-related aversion and could be relevant for the development of treatments for OUD [90, 91].

## Supplementary information

Supplementary Information

## Data Availability

Most data are included in the manuscript and supplementary information and are availabe upon request.
