# Supplementary material for: Naloxone precipitated withdrawal increases dopamine release in the dorsal striatum of opioid dependent men
Source: Transl Psychiatry. 2021 Sep 1;11:445. doi: 10.1038/s41398-021-01548-8 (PMC8410787; doi:10.1038/s41398-021-01548-8)
Supplement: Supplementary file 1 — Supplementary Information [file 41398_2021_1548_MOESM1_ESM.docx]

**Supplementary Material**

**Naloxone precipitated withdrawal increases dopamine release in the dorsal striatum of opioid dependent men**

Ehsan Shokri-Kojori^1*^, Gene-Jack Wang^1^, MD, Nora D. Volkow^1*^, MD

^1^Laboratory of Neuroimaging, National Institute on Alcohol Abuse and Alcoholism, National Institutes of Health, Bethesda, MD, USA.

^*^To whom correspondence should be addressed:

Ehsan Shokri Kojori, PhD; email: [ehsan.shokrikojori@nih.gov](mailto:ehsan.shokrikojori@nih.gov)

Nora D. Volkow, MD; email: [nvolkow@nida.nih.gov](mailto:nvolkow@nida.nih.gov)

**
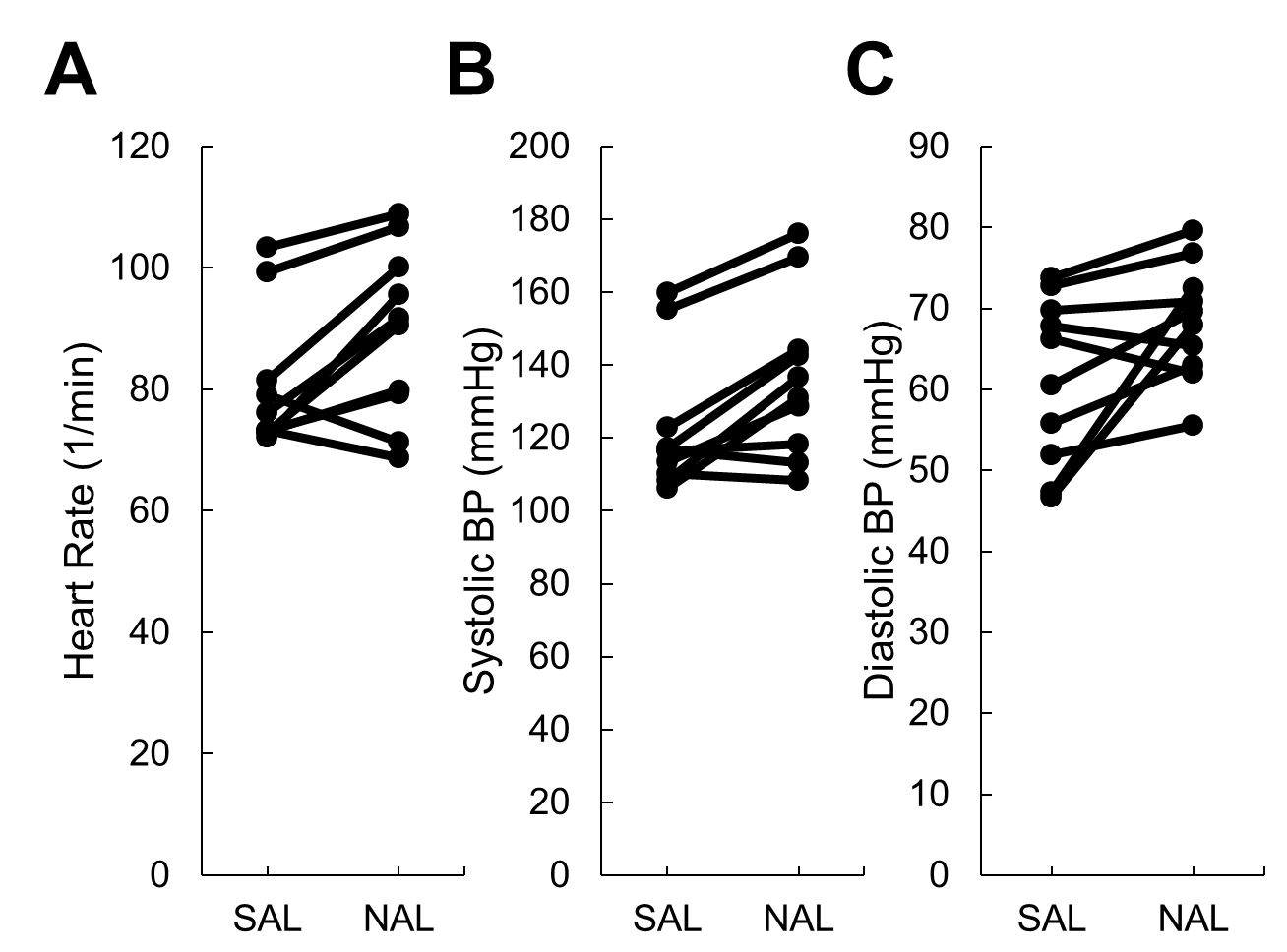
**

**Supplementary Figure 1. Effect of naloxone (NAL) on cardiovascular measures in OUD participants.** **(A)** Changes in heart rate with NAL injection (*p* = 0.04, uncorrected). **(B)** Changes in systolic blood pressure (BP) (**p* = 0.004, *p*_FDR_ < 0.05). **(C)** Changes in diastolic BP (*p* = 0.02, uncorrected). SAL: saline, NAL: naloxone.

**
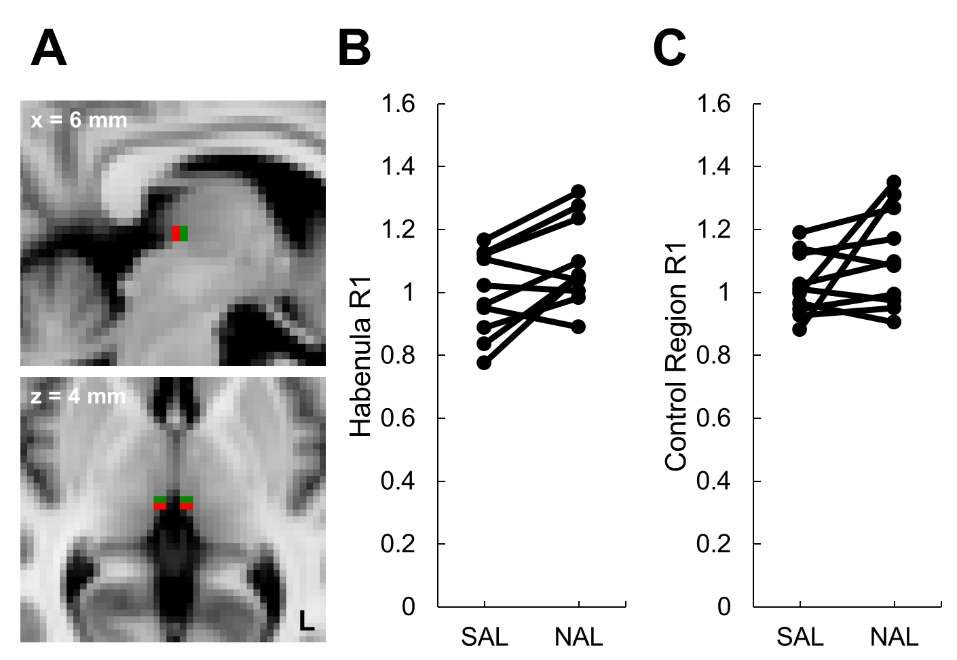
**

**Supplementary Figure 2. Habenula mask and NPW effects on R1. (A)** Red and green colors show the location of bilateral habenula and the control region masks, respectively. **(B)** Effects of NPW on R1 in the habenula (*p* = 0.022). **(C)** Effect of NPW on R1 in the control region (*p* = 0.124). R1 was used as an estimate of relative cerebral blood flow.

**Supplementary Table 1.** Subjective behavioral responses to naloxone precipitated withdrawal. Measures were obtained at baseline (approximately 10 min prior to NAL injection), and 10, 30, and 60 min post naloxone injection. Values represent across subject average (and standard deviation) of measures in each time point (**p* < 0.05, uncorrected; ***p*_FDR_ < 0.05, corrected). For the naloxone withdrawal index, the first principal component of 11 time points (corresponding to 7 behavioral measures) which showed a significant change relative to baseline (*p* < 0.05, uncorrected) was calculated. This component accounted for 37% of variance across these subjective measures where higher *z*-scores were associated with more aversion.

| **Measure** | **Baseline** | | **10 min** | | **30 min** | | **60 min** | |
| --- | --- | --- | --- | --- | --- | --- | --- | --- |
| *Alertness* | *8.6* | (*2.1*) | *7.7* | (*2.9*) | ****7.3*** | (***2.3***) | *8.1* | (*1.8*) |
| *Anxiety* | *1.9* | (*3.2*) | *****6.9*** | (***3.7***) | ****4.1*** | (***3.8***) | *2.8* | (*4.4*) |
| *Annoyance* | *0.4* | (*1.3*) | *****5.2*** | (***4.5***) | ****1.8*** | (***2.5***) | *0.8* | (*1.6*) |
| *Loss of control* | *2.1* | (*3.2*) | **5.1* | (*3.9*) | *3.1* | (*3.5*) | *1.5* | (*1.9*) |
| *Depression* | *1.9* | (*3.2*) | *1.8* | (*3.8*) | *0.5* | (*1.6*) | *0.0* | (*0.0*) |
| *Distressful thoughts* | *1.2* | (*3.1*) | *3.3* | (*4.5*) | *2.1* | (*3.6*) | *1.2* | (*3.2*) |
| *Happiness* | *6.3* | (*2.1*) | *****2.8*** | (***2.7***) | *5.0* | (*2.7*) | *6.5* | (*2.2*) |
| *Mood* | *7.1* | (*1.7*) | *****3.6*** | (***2.7***) | ****5.6*** | (***2.1***) | *6.7* | (*2.0*) |
| *Restlessness* | *1.0* | (*1.6*) | *****6.2*** | (***3.6***) | ****3.9*** | (***3.2***) | *1.6* | (*2.0*) |
| *Concentration* | *8.4* | (*1.8*) | *6.4* | (*3.3*) | *8.2* | (*1.4*) | *8.6* | (*1.3*) |
| *Desire to use opiate* | *6.6* | (*4.2*) | *5.8* | (*5.0*) | *5.5* | (*4.9*) | *3.9* | (*4.4*) |
| *Optimism* | *3.0* | (*2.3*) | *3.1* | (*3.1*) | *2.6* | (*2.2*) | *2.2* | (*2.0*) |
| *Activeness* | *3.2* | (*3.5*) | *3.8* | (*3.5*) | *2.7* | (*3.1*) | *2.8* | (*2.5*) |
| *Indifference* | *7.3* | (*3.2*) | *6.6* | (*4.2*) | *6.8* | (*3.4*) | *6.8* | (*3.6*) |
| *Irritability* | *5.6* | (*4.3*) | *6.4* | (*3.1*) | *6.4* | (*3.7*) | *5.0* | (*4.9*) |
| *Pain* | *0.9* | (*2.0*) | *3.4* | (*2.7*) | *1.8* | (*2.4*) | *0.8* | (*1.6*) |

**Supplementary Table 2.** Areas showing reduced [^11^C]raclopride binding potential in naloxone versus placebo condition in opioid dependent men. All effects survived *p*_FWE_ < 0.05 (cluster-size corrected). Coordinates are in the MNI space (voxel size = 2-mm isotropic).

| **Region(s)** | **L/R** | **Brodmann area(s)** | **Cluster size** | **Peak coordinates  (x, y, z)_mm_** | | | **Peak  *t*-value** | |
| --- | --- | --- | --- | --- | --- | --- | --- | --- |
| **Caudate**  **Putamen** | L | - | 595 | -20 | -6 | 20 | | **7.72** |
| **Caudate**  **Putamen** | R | - | 758 | 20 | 12 | 10 | | **7.06** |

**Supplementary Table 3.** Areas showing increased relative cerebral blood flow measured with changes in R1 (see Methods) in naloxone versus placebo condition in opioid dependent men. All effects survived *p*_FWE_ < 0.05 (cluster-size corrected). Coordinates are in the MNI space (voxel size = 2-mm isotropic).

| **Region(s)** | **L/R** | **Brodmann area(s)** | **Cluster size** | **Peak coordinates  (x, y, z)_mm_** | | | **Peak  *t*-value** | |
| --- | --- | --- | --- | --- | --- | --- | --- | --- |
| **Insula**  **Putamen** | L | - | 606 | -12 | 4 | 26 | | **5.78** |
| **Putamen**  **Insula**  **Pallidum** | R | - | 298 | 40 | 16 | 4 | | **8.84** |
